# Meaning in Psychosis: A Veteran’s Critique of the Traumas of Racism, Sexual Violence, and Intersectional Oppression

**DOI:** 10.1007/s11013-023-09824-6

**Published:** 2023-05-03

**Authors:** Ippolytos Kalofonos

**Affiliations:** 1 HSR&D Center for the Study of Helathcare Innovation, Implementation & Policy (CSHIIP) & Mental Illness Research Education & Clinical Center (MIRECC) Health Services Unit, Greater Los Angeles VA Health System, 11301 Wilshire Blvd, Los Angeles, CA 90073 USA; 2 Center for Social Medicine and Humanities, Jane and Terry Semel Institute for Neuroscience and Human Behavior, David Geffen School of Medicine, UCLA, Los Angeles, CA 90095 USA; 3 UCLA International Institute, 11248 Bunche Hall, Los Angeles, CA 90095 USA; 4 UCLA Department of Anthropology, 375 Portola Plaza, Los Angeles, CA 90095 USA

**Keywords:** Psychosis, Meaning, Veteran, Psychiatry, Clinical ethnography

## Abstract

This clinical case study presents the case of a Latina Veteran experiencing psychosis and draws on eclectic theoretical sources, including user/survivor scholarship, phenomenology, meaning-oriented cultural psychiatry & critical medical anthropology, and Frantz Fanon’s insight on ‘sociogeny,’ to emphasize the importance of attending to the meaning within psychosis and to ground that meaning in a person’s subjective-lived experience and social world. The process of exploring the meaning and critical significance of the narratives of people experiencing psychosis is important for developing empathy and connection, the fundamental prerequisite for developing trust and therapeutic rapport. It also helps us to recognize some of the relevant aspects of a person’s lived experiences. To be understood, this Veteran’s narratives must be contextualized in her past and ongoing life experience of racism, social hierarchy, and violence. Engaging in this way with her narratives pushes us towards a social etiology that conceptualizes psychosis as a complex response to life experience, and in her case, a critical embodiment of intersectional oppression.

## Introduction

Speaking with an individual experiencing psychosis can be confusing. Non-consensual reality and unexpected associative patterns are the hallmarks of psychotic speech. Psychiatrists are trained to listen as a means of categorization to identify the presence of psychotic symptoms—disordered thinking, hallucinations, and delusions—but not to focus on the content of speech that is deemed to be psychotic (McCabe et al. 2002). One psychiatrist characterized this as being trained “to listen. But also not to listen” (Van Meer 2003). It is not the content but the presence of hallucinations and delusions, characterized as “fixed, false, and idiosyncratic beliefs” that are considered relevant (Barta and Rivkin 2017), and as indicative of biochemical, neuroendocrine, neuroanatomical, and genetic abnormalities (Andreason 1985). For mainstream psychiatry, the presence of psychosis points to the need for risk assessment (are the voices telling you to hurt yourself or anyone else?) and treatment with antipsychotic medications in the hopes of eliminating the symptoms. The narrative content and the meaning of the speech of individuals diagnosed with psychosis is not considered relevant (Longden et al. 2012; Corstens, Longden and May 2012; Jones and Shattell 2013, 2016).

This narrow interpretation of narrative and speech as symptoms of brain dysfunction is the outcome of a shifting paradigm of treatment from social, psychoanalytic, and psychodynamic frameworks to a focus on psychotropic drugs as the primary treatment intervention. This most recent turn towards biological reductionism was not driven by scientific discovery, but by neoliberalism and the destruction of the welfare state and public mental health infrastructure, and the rise of direct-to-consumer marketing and health management organizations, leaving psychiatrists with one tool, the prescription pad (Braslow 2021). Psychiatrists have embraced biological reductionism, a view that has increasingly made patients’ subjective lived experiences, life histories, and social contexts irrelevant in the psychiatric encounter. This is not so much because psychiatrists lack curiosity about their patients’ lives. Instead, for most psychiatrists, these experiential and contextual details have become noise that, at best, are epiphenomenal to the signs and symptoms of a disease process that is, at its heart, biological and largely treatable by biological means. While the dominant approach in contemporary psychiatry tends towards this biological reductionism, meaning-centered approaches to psychosis persist in the margins of psychiatry.

In this clinical case study, I share the case of Rosa, a Veteran with whom I worked as a psychotherapist in a West Coast Veterans Affairs clinic. I am trained in a broad range of psychotherapeutic approaches, including cognitive behavioral and psychodynamic approaches. Rosa had been diagnosed with schizophrenia and PTSD prior to our working together. She was referred to me by her psychiatrist, who was frustrated by her refusal to take medication despite spending their monthly appointments trying to convince her to take it. Eventually, she agreed to take the medication, but then she stopped coming to her appointments. She continued to come to our appointments, however, because she appreciated having a place to “vent,” as she put it. She later confided to me that she never took her medications because she thought they were poison and were not going to help her. She did not believe she had an illness that required medication.

Rosa’s psychiatrist documented the presence of psychotic symptoms—tangential thought process, delusional thought content, auditory hallucinations—as well as some of the content of her psychosis—beliefs she was under surveillance, hearing the voices of White women who disparaged her—but, in the spirit of contemporary psychiatry, his engagement with her was focused on medication. Unfortunately, the outcome of treatment dropout is not uncommon, as rates of disengagement with mental health services by individuals diagnosed with psychosis are reported to be higher than 50% (Reynolds et al. 2019). While this is often attributed to lack of insight and treatment refractory symptoms which putatively render an individual incapable of thoughtfully rejecting what is offered, an inability or refusal by psychiatrists to listen, hear, and understand is likely an even more fundamental cause of disengagement (Jones n.d.).

In this clinical case study, I argue that attending to the meaning in psychosis and connecting it to the broader context of an individual’s lived experience and social world is important for not only clinicians and scholars who engage with psychosis, but for social justice as well. The process of striving to understand the meaning and significance of the narratives of people experiencing psychosis is important in developing empathy and connection, which is the grounds for the development of trust and therapeutic rapport. It also helps us to recognize some of the relevant aspects of a person’s lived experience. In Rosa’s case, her narratives are rich with meaning, communicating painful truths about her life. In order to be understood, her narratives need to be contextualized in her past and ongoing life experience. Engaging in this way with Rosa’s narratives pushes us towards a social etiology of psychosis that conceptualizes psychosis as not simply biological anomaly, but a complex response to life experience, and in Rosa’s case, an embodiment of oppression. This makes Rosa’s own political agency visible and her calls for justice and recognition intelligible. She can be heard as, in the words of anthropologist Kim Hopper, offering “critical commentary shaped by [her] own trying times and tuned to the fault-lines of [her] fractious culture” (2008:200).

I draw on eclectic theoretical sources to guide my understanding of Rosa’s narratives, including user/survivor writings on madness and extreme states, phenomenologically oriented approaches to meaning-making in psychosis, meaning-centered cultural psychiatry and medical anthropology, and psychiatrist Frantz Fanon’s notion of “sociogeny.” Since at least the start of the twentieth century, psychosocial disability activists, identified as ex-patients, service users, survivors, and/or persons with lived experience, have challenged reductionist narratives of mental disability and difference, including frameworks that assume mental illnesses are conditions to be eradicated or prevented and that ignore positive dimensions of mental/neurodiversity (Faulkner 2021; Jones n.d.; Bertilsdotter-Rosqvist, Stenning and Chown 2020; Rashad 2019). This movement counters biomedical reductionism with a “social model” that conceptualizes disability as a product of socially imposed barriers rather than as a result of an intrinsic disorder or defect (Spandler, Anderson and Sapey 2015; Beresford, Nettle and Perring 2010; Beresford 2002; Jones n.d.).

One of the accomplishments of this movement has been the development of user-led services and scholarship by “experience-based experts” (Dillon et al. 2013). Many of these writers challenge clinicians and scholars to talk with service users about the history and content of their voices, visions, and unusual experiences and beliefs and to collaboratively investigate the origins, causes, and understandings of the experiences that are called psychosis (Jones and Shattell 2013; Britz 2017). Phenomenologically informed work indicates that to a great extent, voices and other unusual experiences are subjectively perceived as rich, heterogeneous and meaningful; interconnected with identity, culture, and social and real-life events; and inseparable from the self (Rosen et al. 2017; Corstens and Longden 2013; Jones and Shattell 2016; Woods et al. 2015; Clarke 2018). This orientation evokes an ecological framework that situates unusual experiences in the context of a person’s culture, life history, past and current relationships, spirituality, socioeconomic status, etc. (Higgs 2020). Unusual experiences, be they voices, a sense of being watched, telepathy, spiritual communion, etc., might arise around stressors, such as discrimination, loss, or abuse, and are assumed to be personally and socially significant experiences that are informed by and embedded in the external world and that are natural responses to adverse circumstances. These accounts provide a valuable window of insight into a person’s worldview and lived experience (Higgs 2020; Styron, Utter and Davidson 2017). Another way of conceptualizing this is that extreme experiences and beliefs may carry important messages that call for exploration rather than elimination, and that these experiences themselves might be less of the problem than the difficulties individuals have in coping and living with them (Corstens et al. 2014; Jones and Shattell 2013). At least some of these difficulties may be caused or exacerbated by a person’s own responses to their experiences, as well as by the ways others respond to their experiences.

This body of work by user/survivor activists and Mad Studies scholars reclaims expertise over the meaning and significance of the unusual experiences diagnosed as psychosis and recontextualizes them in the social world (Rose and Kalathil 2019; Kalathil and Jones 2016; Faulkner 2021; LeFrançois, Menzies and Reaume 2013; Clarke, Jones, and Mordecai 2022). These critiques challenge clinicians to go beyond understanding these voices as solely symptoms of brain dysfunction, a perspective that frequently invalidates and silences first-person accounts, and instead call for an approach that contextualizes psychosis within robust conceptions of social and psychological worlds. This resonates with meaning-oriented cultural psychiatry and medical anthropology approaches that assert psychiatric diagnosis and biomedicine are themselves cultural products that reproduce and maintain hegemonic political ideologies about personhood and society (Gordon 1988; Comaroff 1982; Taussig 1980). This scholarship centers social and cultural worlds in the understanding of psychosis and shows that the narratives of people experiencing psychosis are fundamentally shaped by culture, politics, and history (Jenkins and Barrett 2004; Kirmayer 2006; Luhrmann and Marrow 2016; Rahimi 2015). In a case series analysis of narratives of individuals experiencing psychosis in Turkey, Rahimi argues that all meaning is political in the sense that it always represents the legitimization of certain associations as more accurate or truthful than others. He further argues that the basic ideas that constitute narratives, no matter how unusual they may be, are the same building blocks of meaning used by the broader culture and society, just arranged in unfamiliar forms.

These approaches that foreground the interplay of power and subjectivity in psychosis draw on a critical embodiment framework that is exemplified in the work of psychiatrist Frantz Fanon (Eromosele 2020). Best known for his writings on the psychology of colonialism and the phenomenology of the colonial subject, Fanon drew on psychoanalysis and phenomenology to argue that the political and the psychic are intimately tied (Robcis 2020). Fanon was critical of the reductionist psychiatry of his day that pathologized Black people when he wrote: “it will be seen that the Black man’s alienation is not an individual question. Beside phylogeny and ontogeny stands sociogeny…let us say this is a question of a sociodiagnostic” (Fanon 2008:4). Fanon proposed this concept of sociogeny to “describe the ways in which social forces and power relations structured language, pathology, and even claims of identity” (Metzl 2009:157). He posited that racism produced an inferiority complex that began as an economic process that was then “epidermalized,” inscribed in the skin and the body (Fanon 2008; Robcis 2020). Fanon’s argument that racist social structures reproduce themselves in the psyches of the people these structures seek to control is borne out in that experiences of racism, poverty, violence, and trauma are indeed linked to the development of experiences diagnosed as psychosis (Anglin et al. 2021; Morgan, Knowles and Hutchinson 2019; Cantor-Graae and Selten 2005; Bourque, Ven and Malla 2011; Bentall et al. 2012).

## Methodology

My approach is a form of clinical ethnography. As a dually trained anthropologist and psychiatrist, I see psychotherapy as a practice that is both clinical and ethnographic, in that each person’s inner world can be conceptualized as a unique culture with its own history, language, values, and symbolic systems (Lester 2022). Engaging in therapy with an individual entails an ethnographic exploration of that individual’s inner world, a process that takes time, patience, and development of trust. Working within a critical medical anthropological framework, I understand explanations of pain and suffering as potentially associated with the larger social, political, and cosmic orders, and attempt to bridge the “micro–macro, Marxist phenomenologic dichotomies” (Scheper-Hughes and Lock 1991, 1986:137).

The material shared in this case study is from my interactions with Rosa and her family members as Rosa’s therapist within the context of clinical encounters that took place over two and a half years. While most of our encounters occurred within the clinic, I visited Rosa and her mother at home on several occasions at Rosa’s suggestion in order to include her mother in a few therapy sessions. I also draw from Rosa’s medical records, which extend back to her military service time, an unusual resource in the context of the fragmented US health system, and one which allows me to triangulate between contemporary and past narratives and to follow events through the words of past medical providers. I wrote down notes about each session after it concluded, which were often summaries of conversations, direct quotations that I remembered, and my own impressions and reflections. I also audio recorded many of our sessions, with Rosa’s permission. This case analysis draws from these notes and recordings. Anything presented as a quotation is either from an audio recording or from notes that were written immediately after the session.

Philosopher José Medina points out that treating speakers as informants does not necessarily avoid silencing and objectifying, as “their voices can still be constrained and minimized, and their capacities as knowers can still be undermined” (Medina 2012:204). Rosa is my informant/patient, and her epistemic subjectivity and agency are limited by the fact that she is not the author of this text. I present Rosa’s narrative in her own words, but I provide my own understanding and interpretation of her words based on my relationship with her and my knowledge of her life experience. Powerful first-person accounts of unusual, non-consensual experience do exist (e.g., Clarke 2018; Britz 2017; Saks 2008; Longden 2013; Wang 2019; King 2007; Beresford and Russo 2022). Rosa is not likely to publish her own first-person account, yet her experience and words nonetheless merit broader consideration. Rosa told me on several occasions that she sought justice for herself and her family, to be seen, recognized, and understood on her own terms. I present my interpretation as a contribution towards a form of justice, limited though it may be, for Rosa and others like her who experience psychosis yet have few opportunities to be heard. She has given me verbal and written permission to write about her case, though I have anonymized all people in this account and altered key personal characteristics to preserve confidentiality. Rosa has not read this manuscript. She moved out of the area before it was completed and my attempts to contact her have not been successful.

This paper is structured to recreate for the reader my own experience with her, confronting her at times confusing, apparently contradictory narratives before contextualizing them within her life experience. I begin with her chart history that I accessed prior to meeting her. I then share some of the narratives that she discussed with me and my own impressions of those. Her life history follows, mostly from her perspective as she tells it, but supplemented by her mother’s perspective, and by additional history from her chart.

## Current Presentation

Before I met Rosa, I could see in her chart that she had diagnoses of schizophrenia and PTSD. She was 100% service connected for the PTSD that she suffered while in active duty, which made her eligible for subsidized medical care at the VA and a monthly payment of around $3000. She had a documented incident of military sexual trauma, “MST.” She had a history of dropping out of clinical care with psychiatrists, therapists, and primary care providers alike. As indicated above, she saw another psychiatrist for medication management, but she never took the medication she was prescribed and eventually terminated her relationship with that psychiatrist but continued to see me until our clinic became virtual during the COVID pandemic. She preferred to wait until we could meet in person again rather than having virtual therapy sessions, but then moved out of the area before our clinic re-opened to in-person visits.

A recent clinical intake documenting her current presentation as well as her history noted she was friendly though “heavily digressive and hyperverbal [with] persecutory delusions. Endorses frequent monitoring…concerns over racial identity, vile criminals who her mother used to work for. Emphasizes repeatedly that she’s never done anything wrong, never murdered anyone…Says she feels ‘sad all the time.’ Adamantly denies SI/HI… Says she was discharged from military because ‘they thought I was sleeping with everyone.’” The history section documented an “unremarkable childhood/developmental history…had some close friends.” She was born in Central America and migrated to the US at age 8. It also noted a 10-year marriage with a physically abusive husband that ended in separation and 3 spontaneous miscarriages. She lived with her mother, with whom she felt close, but “the voices want us to fight all the time.” She was unemployed and a typical day consisted of “wandering around.” She was described as “hostile/threatening” and “unusual/bizarre,” with “inappropriate giggling,” “labile affect,” and “angry mood.” The note concluded that the “constellation of disorganization of thought, paranoia, and delusional ideation are likely consistent with a diagnosis of schizophrenia.” There was no attempt to connect her presentation with her history in this note, or to make sense of her presentation beyond diagnosis of schizophrenia and treatment with an antipsychotic medication. I would learn many more details of her history and the ways it did relate to her experiences.

When I met Rosa, she was middle-aged and in good health. She dressed casually, in jeans, t-shirts, and sweatshirts. She was engaging and animated when she spoke. At times she did get angry or tearful, and at other times laughed. These emotional responses did tend to be related to the content of what she was talking about. She often wore t-shirts of heavy metal and punk bands. She told me “these are the bands from the sunset strip. That’s my scene.” Rosa lived with her mother and her 2 birds in a subsidized studio apartment. She spoke Spanish with her monolingual mother, but she spoke fluent Standard American English with me. She watched a lot of movies and television and often referenced celebrities and British royalty. While she spent a lot of time at home, she went shopping and took excursions to the beach and around town. Every so often, she took trips that were regional, national, and at times international. Sometimes she would visit the sites of US army bases where she was formerly stationed as well as pop culture landmarks.

At our first session, she asked me to help her get a military lawyer because of what happened to her when she was in the military. The government told her that she was raped but this was not true. She went on to explain that a woman was raped, but it was not her. It was a “Hispanic girl” but Rosa explained that she is German. Furthermore, she maintained that “those guys were Swedish,” referring to the assailants. The military put her face on this woman’s face and made it look like she was actually raped using special effects. She wanted to clear her friends, with whom she had consensual sex, and she wanted the truth from the government and justice for the girl who was raped. She didn’t get help. Rosa maintained that she never was raped herself. She dated men “of my own race.” Rosa clarified that here, she was referring to her blood type, which she viewed as something more authentic and accurate than skin color. Rosa told me she had a “German ear,” so she heard “further than normal.” She heard voices that were sent to her via a technology the government used to control people, but “any idiot can use it.”

In that first session, Rosa identified some of the major themes that persisted in the narratives she continued to share with me: the need to address injustice; sex and morality; race, blood, ethnicity, and identity; gender and violence; and underlying all these themes, the questions of safety, security, power, and truth. I asked her if her “MST” status referred to that putative rape, and she again denied being raped herself. When I asked her what justice might look like, she said getting help for the girl who did get raped. I wondered at that time if she was referring to herself, as if she wanted help but could or would not admit it.

### Voices, Morality, and Judgment

In the military, she explained, “they make you hear voices…They pushed me to the limit and broke me, that’s when I left…They made this thing up about being medically retired.” She heard many voices, too many to count. She often likened her voices to hearing different radio stations all at once, and she tried to focus on the one that was the least bothersome. She experienced these voices as overhearing ongoing conversations, at times they talked about her and at other times to her. Sometimes the voices revealed truths about the world, about her family, about herself. Some of the voices were of “ordinary people” often having conversations, some of “Nordic Nazi women, White trash.” These Nordic Nazi women were connected to the women with whom she attended high school, as well as women from the military. These were the most distressing of the voices. They were derogatory, often commenting on her body and her behavior. In particular, these Nazi women she heard:

judge me for being a slut. But there is nothing wrong with sex! The Nazis have made us all think sex is bad and shameful. It is normal and there is nothing wrong with it…These people are probably hearing the sins of their lives, murders and fraud. I’ve never done that! I have no guilt, sex is not something to feel guilty about. These people think I’ve done wrong, sex is not wrong! Sex doesn’t kill!

Rosa denied there was anything wrong with any of her sexual experiences or anything she had done at all. Once while we were discussing emotions she experienced, she vehemently denied experiencing shame or guilt. She added that it was the Nazis who should feel shame and guilt, not her.

She believed many people were controlled by voices, including her family, but few were aware that it was happening. At one time the voices impersonated her mother and one of her uncles, but she ended up figuring out it was not her mother because the voice was talking about computers, about which her mother knew little. She could not always tell when the voices were lying, however. Most of the time, she said she would be happier without the voices. On one occasion when I asked her if she wanted to silence the voices, she said no, because they reveal the truth about things. Hearing them helped her protect herself: “I trusted everybody but it hurt me.” The voices did sometimes protect her or warn her, but more often they threatened and criticized her. Most of the time, Rosa spoke with contempt about the voices. She felt they controlled everything about her life and wanted to kill her. She had no choice but to fight back, but she could not find any effective way to do so. When she applied for jobs, they seemed to intervene, sending an imposter to the job interview and submitting phony resumes and applications in her name, so she stopped looking for work. She was also convinced that the people she interacted with heard bad things about her, so she was unable to meet people or make friends and was left alone. This is why her “boys in the military,” as she referred to them, had to have sex with her while she was passed out, which she assured me was consensual.

### Race, Identity, and Blood

Rosa once told me: “I heard a strange thing; about how White people have been living as Black. Remember that book, *Black Like Me*? There are lots of White people living as Black.” This was a book she read in high school, published in 1961 by a White journalist, John Howard Griffin, who dyed his skin Black in order to experience the segregated South as a Black man (1962). She was disturbed by the fact that White people could paint their skin Black or Brown: “you trust another person because they are Brown, but really they might not be…*are there,* any Black people?” She had recently returned from a trip to the southern United States and she told me she saw a lot of Black people, but she thought the majority of them were White Nazis painted Black.

She talked about race as being accurately defined through blood type rather than skin color. The Nazis were blood type B and wanted to eliminate her blood type, O. She sometimes connected this blood type to her own royal lineage. She felt she was being persecuted by White Nazi women because her family was heir to the fortune of Louis the 14th: “they want the castles but they belong to us and they can’t get them as long as we are alive. They want us to die.” While at times she matter-of-factly identified as “Hispanic,” at other times she said she was German. She clarified that Germans were unfairly and incorrectly associated with Nazis. At the same time, she trusted people of color more than White people, with comments such as “there is no such thing as a dark terrorist. I had no idea that the majority of White shit…is doing the harassment. It has nothing to do with the beautiful people of Iran and Iraq, I know the beautiful people. It has nothing to do with Brown skin, either.”

Rosa shared that many people claimed to be related to her, but she only identified her mother and one uncle as family. The others were really Nazis in disguise. They wanted to drive her away from her mother and uncle, whom she loved. She invited some family members to therapy with her in the hopes that they too might tell us about the voices they heard. But they did not. Despite frequent conflict and disagreement between them, Rosa told me she enjoyed therapy with her mother, as it gave them something they could do together. She also brought two of her uncles on separate occasions and once together. When I asked her what she hoped to accomplish by bringing her family to therapy, she said, tearfully, “I want us to be identified. It’s just us 3 [herself, her mother, and her uncle]. I want people to see us. We are ugly because royalty needs to be White. I just want people to know who we are. It’s just us 3.” Rosa’s mother, an evangelical Christian, felt she could not tolerate all the bad things Rosa said. She was certain Rosa was negatively affecting her health. Rosa’s mother had multiple chronic illnesses and used a cane and a wheelchair to get around. She wondered how long she and Rosa could tolerate living together, yet at the same time worried about Rosa and wanted to support her.

### Class, Work Ethic, and Exploitation

Rosa was frustrated at not being able to afford urban life. She and her mother both wanted leave their cramped studio apartment, but they could not afford anything else. Rosa wanted to move to another state, where she once was stationed, but her mother did not. One uncle talked of buying the family a ranch in Central America, but Rosa rejected this fantasy: “there is nothing for me there. My life is here.” Eventually, Rosa did move by herself to that other state.

Rosa noted that she had worked all her life, yet she was powerless and poor, and the Nazis did not work at all, yet were powerful: “I’ve been working, they are living off of us, living off of other people. They are the overbreeding garbage of the world….they’ve never done anything useful.” She used to think her former classmates who had homes in the hills all gained their wealth through hard work, but later she became convinced they stole it all: “The hills are full of squatters who don’t pay rent or bills and live off of everyone who is working honestly. There are so few of us [who work honestly], so many of them. That’s the real sin, not sex.”

### Safety, Fear, and Surveillance

At times, Rosa was scared to leave the apartment lest someone enter and poison the birds or her mother. She suspected her elderly mother’s health problems to be the result of poisoning. On the other hand, she worried her mother was an unwitting accomplice of the Nazis, and for a period would not let her mother stay home if she were not also home with her. Rosa and her mother fought a lot, though Rosa blamed this on the Nazis turning them against each other. In order to feel safe, she changed the locks on the door of their apartment several times. She was pretty sure everything was being video-recorded and watched by the Nazis and she did not want to be seen, so she showered in the dark and isolated at home. The idea that they were watching everything also made her uncomfortable to write anything down. She did not make grocery lists and did not tell anyone what she planned to buy or where she was going. Once when I presented her with her answers to a brief questionnaire she had completed a few weeks prior, she recoiled and she asked me to put it away. She told me she recognized it as her own writing, but knew she did not write it, and she found this disturbing.

I asked Rosa what she wanted out of life:

I don’t even know anymore. I wanted to have a cottage, marry, have kids, but the Nazis don’t want me to have that. I went through such a difficult time. They wanted me to be homeless. They are trying to take my monthly payment away…I want love, but they won’t give me that. If I could redo my life, I would do it the same way, I’ve been loved and I have loved deeply. They don’t want me to have children….But the world they created is very strange. I never knew we lived in this Nazi regime. We need a Nazi regime book for dummies or something. I want to know what everything means. When somebody asks me for directions I get freaked out because they mean something else. They know somebody’s always watching so they ask a certain question to give White trash a clue of something. They use you as a way of communicating with this disgusting White trash. So every time they come to me and say oh, where such and such is, I hate it, I don’t want to respond I just want to walk away. I had no idea that this is the kind of world that they wanted us to live in, you know? It’s very, strange to me to deal with it. Is there a world for us? I don’t know how to be anymore or how to react with people.

She was bewildered by the injustice she was experiencing and felt she did not know how to behave anymore, that the world was not what she once thought it was and it was unclear where she fit within it. She was certain her ignorance about computers was being used against her. But despite her ignorance she asserted she did not deserve persecution because she had never done anything wrong:

I don’t know anything about technology or computers. All I know is how to turn on my computer and watch movies. I don’t know anything about it. I didn’t do so bad for not knowing that this existed because I have never behaved like these disturbed people. I have never killed anybody, I have never committed fraud. I have never hurt anyone but myself. The worse I have ever done is drive drunk.

She asserted that she was moral and blameless, but the voices:

tell me that sex isn’t moral and that is why I talk about sex…I was born with nothing, and I was still taught to do the right thing. I don’t know how that is. I didn’t have paper money, we didn’t eat, we were pretty much vegetarians when I was a kid. Because we didn’t have anything. And yet in this place with no running water, no electricity, the Nazis were still watching. And they still put their disgusting White trash around my grandfather….I want these people to get caught. I don’t want these people to get away with this.

She once commented: “why won’t they give me a note telling me what they want? They ask me to do so many stupid things! Move this, put it there then put it there. They want power.” I asked her if she’d like to exercise more power over her life, over the voices, she said yes. She wondered “if they don’t like me, why do they watch me? They have kept me trapped my entire life.”

### Life History

Rosa was born in Central America in a region mired in US-aided civil wars. She lived with her grandparents, whom she adored, and extended family in a small house with a dirt floor in a rural village. Her mother lived and worked several hours away in the capital city. Her father left the family when she was 2. Despite this poverty, she says: “I had a great childhood. I was able to be a kid.” She and her uncle laughed together when they recalled playing together when she was a girl and he a young man.

But she also described darker events. People poisoned her grandparents’ animals because “they knew who my grandfather was. He was born in eastern Europe and was a descendant of European royalty. Crimes were committed against him by Nazis who were Norwegian. He was left penniless. Our blood says it all.” Her mother used similar language in explaining that Rosa’s grandfather was of European “blood” and had a German mother and Spanish father, so he was White-skinned. Many of their relatives were also White with green eyes. Her side, however, was “*indio, morenito*”—indigenous, Brown, like she and Rosa.

Rosa told me she had her first sexual experience in Central America at age 7. A family friend, whom she called uncle, attempted to have sex with her and told her it was a part of first communion. Laughing, she casually explained they did not end up having sex, but insisted that this was a normal part of first communion. “I think I would instigate it,” she reflected. Separately, her mother confirmed Rosa was sexually abused as a child, when she was away, by a family friend, but they had never spoken to each other about it.

When Rosa was 8, she and her mother moved to the US. Her first elementary school was predominantly Spanish speaking, which helped ease a difficult transition. After her mother found work cleaning houses in an affluent neighborhood, however, Rosa transferred to public school there, where very few spoke Spanish: “I wasn’t meant to have any friends. Any time I had a friend, they were targeted by Nazis.” She recalled her classmates were “children of terrorists and the Gestapo…I went to school with the worst terrorists.” She described herself as “a loser” in high school, “a nobody.” She did have a best friend whom she described as “a Russian Jew,” and whom she was very close to. Her friend’s family would take Rosa along on family vacations. Rosa referred to her friend’s mother as her second mother.

Rosa worked part-time at a mall during high school and fulltime after graduation as a receptionist for several years. She described an active social life during her twenties, enjoying music and dancing. She felt so ashamed she was “only” a receptionist and rented an apartment, however, that she did not go to her ten-year high school reunion, despite wanting to attend. Around the time of her ten-year high school reunion, Rosa enlisted in the military. This was soon after 9/11 and Rosa was in her late 1920s. She was motivated by a desire “to do good.” She also was concerned for her livelihood, however, and thought after she got out of the military she would never be unemployed: “I wanted something secure, to help and get help.” Her mother recalled that Rosa wanted to serve the country and then to go to college. She told her mother she wanted to buy her a nice house someday.

Rosa was sent to Iraq, which she described has having been “tense…we were all innocent, we didn’t know about technology, voices. We must have been stronger than we thought.” She worked in the dining facility of a Forward Operating Base and also drove convoy trucks. These engagements put her in close proximity to mortar fire. She recounted: “I thought I’d be reading to Iraqi children. I didn’t realize it would be red against red, I’d be fighting my own people for these White Nazi shits.”

Shortly after enlisting, she met a man, a fellow service member who was White and had a German surname, whom she eventually married. He cheated on her with other women (whom Rosa has identified as White), was abusive, and they both drank heavily. Rosa once told me, however, she was “grateful to the men in the military, for what they did. I had children. They did their job as men.” Her lone pregnancy, however, ended in a miscarriage. She was devastated by this and wanted to die at the time. She tried to drink herself to death and recalled, “I didn’t want to feel anything anymore.”

Putting Rosa’s account alongside her family’s and her medical records allowed me to piece together the following chronology: she enlisted soon after 9/11with no psychiatric symptoms. She was deployed shortly thereafter. She suffered a miscarriage that was emotionally devastating and resulted in her first psychiatric hospitalization, which lasted several weeks. She was married and became a US citizen, but this was an abusive relationship marked by violence and infidelity. During a period of separation, Rosa was raped at her own residence by 3 male service members, one of whom she was dating, after she had passed out from drinking. The assailants, all African-American, took pictures and shared them on social media. They were charged, tried, convicted, and imprisoned. Rosa was diagnosed with PTSD resulting from both this incident as well as her proximity to mortar fire in Afghanistan. In the months following the rape, military clinicians documented Rosa’s shame and anger surrounding this event. She suffered “anxiety,” “depression,” and “paranoia” about being followed and watched by “Hispanic and African-American men.” Nearly a decade after enlisting, she was medically discharged from the military because she could “no longer fulfill her duties as a service member,” according to her discharge paperwork. Following military discharge, Rosa moved back in with her estranged husband, but the abuse continued. They separated and she moved in with her mother. Auditory hallucinations were documented for the first time soon after, during the year she last worked. She attended community college classes for a couple years but dropped out. She had one postmilitary hospitalization after this.

### Analysis

While her clinicians and family members dismiss her narratives as delusional, lacking insight, and untruthful, I argue a close analysis of Rosa’s narratives provides valuable insights into many aspects of her own lived experienced and her own suffering. They emphasize the savage brutality and strangeness of a world fractured by racism, sexism, inequality, exploitation, and violence. On one level, Rosa’s story is a case study of the toxic social experiences that the epidemiological scholarship indicates facilitate psychosis. Experiencing multiple childhood traumas, particularly sexual abuse, as Rosa did, is associated with psychosis to an extent comparable to the association of smoking with lung cancer (Bentall et al. 2012). Migration is also associated with increased risk of psychosis, particularly for non-White migrants from poorer countries to majority White countries, as was the case for Rosa and her mother (Cantor-Graae and Selten 2005; Bourque, Ven and Malla 2011; Morgan et al. 2010; Morgan, Knowles and Hutchinson 2019). Poverty, which Rosa also experienced throughout her life, has long been associated with higher rates of psychosis, and evidence is emerging that poverty precedes the onset of psychosis rather than the other way around, as posited by the “social drift” hypothesis (Morgan, Knowles and Hutchinson 2019; Harrison et al. 2001). Rates of psychosis are higher among minoritized ethnic groups where they form a smaller proportion of the population, in a dose–response relationship (Susser et al. 2008; Morgan, Knowles and Hutchinson 2019; Boydell et al. 2001). Rosa spent much of her life in the US as a minoritized individual, and particularly described her experience of attending school in an affluent neighborhood and of the neighborhood where she was residing in these terms, where in both cases she experienced being one of the only people of color. Experiences of perceived discrimination are also associated with psychosis (Oh et al. 2014; Anglin et al. 2021), and Rosa described feeling discriminated against almost constantly. While in her chart it appears that the rape she suffered in the military precipitated her feelings of fear and insecurity that now pervade her daily existence, she was rendered vulnerable to this experience through multiple life events. Rosa’s military experience by itself entailed several traumatic experiences—rape and the subsequent public humiliation, domestic abuse, miscarriage, proximity to mortar fire, loss of her military career due to subsequent medical discharge, divorce, and then a sense of abandonment following separation from the military as she struggled to reintegrate into civilian society when she returned home. This resonates with Fanon’s argument, stressing the structural link between the psychic and the political and that war is a “breeding ground for mental disorders.” In his case series entitled “Colonial War and Mental Disorders,” Fanon wrote “clinical psychiatry classified the various disorders presented by our patients under the heading ‘psychotic reaction’…we believe that in the cases presented here the triggering factor is principally the bloody, pitiless atmosphere, the generalization of inhuman practices, of people’s lasting impression that they are witnessing a veritable apocalypse” (Fanon 1963:183; Robcis 2020).

Rosa’s fractured narratives, presented above, recount the truthful and accurate story of her subjective perspective, but recognizing it requires listening and contextualization of her narrative within her lived experience. Sadeq Rahimi’s metaphor of an earthquake for understanding the impact of psychosis on subjective experience can be helpful in unraveling Rosa’s narratives. Just as an earthquake destroys complex structures but may leave foundational building blocks intact, the experiences leading to psychosis might be thought of as tremors that destroy highly complex structures of meaning, leaving foundational building blocks that function as key points of reference (Rahimi 2015). The earthquake metaphor would seem to fit Rosa’s trajectory, with the military rape and subsequent distribution of humiliating pictures on social media serving as the tremor that ultimately fragmented structures already rendered vulnerable by the many traumas she had lived through. Certain structures were clear for Rosa—the salience of race, gender, and class—yet she struggled to piece together and order a chaotic psychic landscape and to find a space of safety and security. Rosa’s narratives are so dense with meaning and associations that I cannot do justice to them all in this space. The following discussion is my attempt at piecing together the foundational building blocks Rosa shared.

### Identity, Self, and Other

Class, gender, race, ethnicity, and blood are all prominent in Rosa’s narratives, in complex, fragmented, and inconsistent ways. Rosa, marginalized by virtue of her low income and foreign-born and non-citizen status, was drawn to enlist in the military in part by the promise of upward mobility and security, a common path into the US military from communities of color and from low- and middle-income levels. As anthropologist Erin Finley observes, “even in an all-volunteer force some volunteers are more voluntary than others” (Finley 2011:15). This dynamic, in which those who have their opportunities restricted by virtue of their race, ethnicity, or income level are more likely to enlist in the US military as a means of socioeconomic advancement, thus putting them at increased risk of injury or death, is a clear example of structural violence, a process by which prevailing institutions and social structures differentially constrain agency and inflict harm in their routine and everyday operation (Galtung 1969; Pérez 2015; Farmer 1996). Rosa did become a citizen during her time in the military, perhaps taking advantage of fast-track options into citizenship offered by the military (Military OneSource 2020), but she never attained the middle-class status she desired, symbolized by a nuclear, homonormative family and home ownership. Her sense that Nazis were preventing her realization of this status points to a feeling that she was being systematically obstructed, which will be further discussed below.

Rosa believed blood, not skin color or outward appearances, accurately reflected race. Perhaps this is why, despite her appearance as, in her mother’s words, Brown and indigenous, she identified with her grandfather’s German heritage. She also kept her ex-husband’s German surname. This identification as German may indicate an internalization of racism and a desire to identify with the hegemonic White Euro-American status. This resonates with Fanon’s analysis of racist societies that push people of color to view themselves as inferior to the dominant White ideology, resulting in an internalization of an inferiority complex (Fanon 2008). The rigidity of Fanon’s categories is belied by the ambivalence Rosa communicated about race, as despite her identification as German, she also identified as Hispanic and felt she persecuted by Nordic Nazis who had the power to watch her and directly intervene in her life. This Nazi presence might be the embodiment of the White supremacy that thwarted Rosa at so many turns in her life. She felt excluded by her predominantly White and affluent high school classmates (“children of the Gestapo and the worst terrorists”) and teachers and continued to feel excluded by mainstream society, which she identified with the illegitimate squatters in the hills. She lamented that she and her family were “ugly because royalty is supposed to be White,” acknowledging a White supremacist esthetic hierarchy they were subjected to. She expressed a conflicted kinship with both her fellow American service members and Iraqis, as she similarly identified as a victim of White terrorism. She expressed this most powerfully in her assertion that “there is no such thing as a dark terrorist” and the intimation that it was not the “beautiful people of Iran and Iraq” who were the terrorists but “the majority White shits.” This speaks to the “compounded alienation and marginalization” that many non-White solders are subjected to “as they fight in the service of a state that marginalizes them” (Macleish 2013:23).

Rosa has been harmed by individuals of all races and colors—sexually abused as a child by a family member, ostracized in the US both while growing up and after her discharge from the military, physically abused by her White husband and fellow servicemember, and then raped by fellow servicemembers of color. The solidarity she asserted with other people of color, especially Black people, was complicated by this rape. Her military records indicated that in the wake of the rape, she felt fearful around men of color. Her own reconfiguration of race around blood and painted skin may be an attempt to preserve a desired solidarity with people of color as well as her German identity and her desire to be included in White society. Rosa’s fears that she was under surveillance not only reflect the objective reality of the loss of privacy resulting from new technologies and social media (Gold and Gold 2012), but are also echoes of the humiliating pictures of her that were posted online by her assailants in the military.

Rosa insisted that both the childhood sexual event and the documented military rape were consensual. On several occasions, I expressed concern that neither of these scenarios sounded consensual or appropriate. She insisted nothing was wrong with any of the sexual encounters she had experienced, asserting her agency, autonomy, and consent even as a seven-year-old and as an adult when her “boys in the military” had sex with her while she was passed out. While people who have experiences trauma often minimize the impact of painful events, Rosa insisted she was not a victim of male assailants in the military, but rather was a victim of White Nazi women, of White supremacy. She felt judged by the voices that were telling her sex was wrong and she responded by denying there was anything wrong with any of the sex she had ever had, seemingly excusing those who assaulted her. This denial may have shielded her from her own guilt and shame associated with these events. This latter emotion, shame, ties many of Rosa’s painful experiences together and is one she vehemently denied feeling. Shame, the feeling there is something flawed, bad, or worthless about oneself, is so prevalent among people who have experienced trauma and hear voices, that it is considered by some to be particularly associated with the development of voice-hearing (McCarthy-Jones 2017:146–152). Yet, her assertion also recalls anthropologist Rebecca Lester’s discussion of “ambivalent agency” in trauma, which points to an individual’s complex relationship to trauma involving choices he or she made freely (2013). Rosa’s refusal to see herself as a powerless victim may itself be a strength and could reflect her struggle and desire to assert herself in the face of the overwhelming oppression she faced.

Rosa attributed the structural violence that constrained her agency and rendered her vulnerable to physical and psychological injury to the work of Nordic, White, Nazi women, who had long persecuted her family. Who could these women be? She described growing up as one of the few native Spanish speakers in the schools she attended. Her best, and by her account only, true friend in high school was Jewish. She also described her ex-husband cheating on her with White women while in the military. Her disdain for the women who tormented her was matched by her disdain for women who were Veterans and clinicians as well. She refused to see female clinicians. When I referred her to group therapy, she preferred attending a male group rather than a female group. The military is a profoundly masculine domain to the extent that women soldiers—who constitute nearly a sixth of all active duty soldiers—are deeply invested in the masculine homosociality and often profess negative stereotypes of other women soldiers (Macleish 2013:18–19). The fact that Rosa was tormented by female voices and identified with males may indicate the internalization of the gendered hierarchy of the military that values masculinity over femininity. When I asked her what she made of the fact that it was all women that seemed to be tormenting her, she told me it had always been women who had gotten in her way and tormented her. At one point she brought me a list of names of women with whom she had attended high school and served in the military who were implicated in this.

Rosa was trying to figure out who she was and was not connected to and how. She felt under constant threat and was conflicted about who to trust and who to identify with. She disavowed all of her family at some point, including her mother. Her family was never able to adequately protect her, neither when she was a child, nor as an adult. While she lamented her childlessness and the judgment she heard from Nazi women regarding her own sexuality, she characterized the Nazis as overbreeding. She felt the Nazis sought to eliminate her blood type because of her family’s royal lineage. This was an active genocide as she wondered if there were any people of color left and whether they had all been replaced by painted White bodies. She connected this to the whitening of the neighborhood where her mother had lived for twenty years. This neighborhood was, in fact, considered one of the most rapidly gentrifying areas of the city.

Rosa did not always agree with my interpretations when I shared them with her, and I do not claim to have facilitated any dramatic clinical breakthroughs. We did both agree on many interpretations—that she was profoundly lonely, for example—and we sought ways to remedy that beyond our weekly meetings. I do believe that the process of trying to understand her narratives on her terms was the basis of my ability to empathize with her and to see her as someone who has been hurt rather than someone who is damaged. This enabled us to work together for over two years, much longer than Rosa typically engaged with clinicians in the past, and I suspect it was because she could feel that empathy, connection, and concern.

## Conclusion

Despite the literal “untruths” her mother and uncle object to, Rosa spoke to many clear truths—her family’s inability to protect her, the structural violence inflicted on people of color and immigrants by White supremacy, the epidemic of military sexual trauma, and the corrosive hypocrisy of American empire which framed Rosa’s life, from her impoverished upbringing in a region embroiled in US-aided military conflict, to her immigration to the US, her enlistment in the army following 9/11, her deployment to Iraq, her medical discharge from the military, and her postdeployment disillusionment. Her military experience involved multiple forms of violence including intimate partner abuse, miscarriage, alcohol abuse and self-inflicted harm, rape, and a perhaps not fully articulated realization that Rosa was fighting on the side of the imperialist oppressor and thus contributing to the entrenchment of the power hierarchy that hurt her and her family. In many ways it was the military experience that seemed to get under Rosa’s skin and lead to a profound alienation. Like many Veterans, Rosa struggled to adjust and reintegrate into civilian society. She continually protested that in the face of these injustices, her own faults and misdeeds, about which her voices berated her, were relatively insignificant. Others committed the crimes that caused her suffering, yet she seemed to be the one being punished. The injustice of poverty and inequality were very clear to Rosa, as the honesty, hard work, and loyalty she and her family embodied never brought them the American dream. Her own labor seemed to not enrich her but the elites living in the hills: “I’ve been working, they are living off of us, off of other people.” Rather than providing stability, her military service left her profoundly insecure, arguably worse off than she had been prior to enlisting. She feared that even her service-connection benefit was under constant threat.

In the face of the tragedy marking her life, Rosa refused to be defeated. She channeled a righteous anger towards the White supremacist forces embodied in the Nazis who seemed to target and persecute her and the family she recognized. She insisted her family bore an unacknowledged value and honor, as persecuted royalty. She escaped a toxic marriage and overcame alcohol abuse. She cared for her pets and her mother, though her ambivalence towards her mother and their constant close quarters led to conflict, and she asserted her independence by traveling and pursuing a better life elsewhere. She was proud to be a Veteran, enjoyed meeting and socializing with other Veterans at the VA, and identified aspects of her military career from which she benefited. But, during the time we worked together she was profoundly isolated and admitted that she didn’t have many extended conversations outside of therapy. She reflected: “I can’t believe I once had a best friend and dated.” Her trips to military bases where she was formerly stationed around the country seemed like attempts to recover her shattered dreams, to make sense of her past and maybe recapture and rewrite it. Ultimately, she acted on this desire for a new beginning in a familiar setting and she moved back to an area where she had been stationed.

Dismissing Rosa’s narratives as incoherent products of a broken brain with no basis in reality is a form of epistemic injustice, and risks obscuring the real traumas she endured and denying the inequalities and power differentials that harmed her in real and lasting fashion. Reducing her narrative to an incoherent collection of symptoms that must be medicated away is a form of blindness, an inability to see what matters. Recognizing the meaning, truth, and reality in her story, on the other hand, helps us recognize Rosa’s own insights into her painful and contradictory lived experience, and moves the site of pathology from a chemical imbalance within Rosa’s brain to a power imbalance in the social environments that have structured her life and that have exerted effects on her psychology and biology (Selten and Cantor-Graae 2007; Anglin et al. 2021). We can reframe her voices from symptoms of a disorder to reminders of enduring pain. The implicit question shifts from “what is wrong with you” to “what happened to you” (Longden 2013; Higgs 2020). Striving to understand what happened to Rosa allows us to learn from her and to connect her personal narrative to structural factors and to recognize the interconnection between biological, psychological, social, and political dimensions.

This directs our attention outward, from an exclusive focus on brain pathophysiology and individualized symptoms and pathology to relationships between clinicians and our patients and between our patients and the broader world—significant others as well as institutions like the military, schools, health systems, and states. Such a perspective begins to acknowledge the layers of meaning contained in psychotic narratives and points us towards the roots of much suffering in violence, poverty, racism, abandonment, and disenfranchisement. It provides a corrective to the contemporary psychiatric commonsense that ignores both the content of psychotic speech and the subjectivity of the person experiencing psychosis, compounding alienation, and exacerbating ongoing suffering and exclusion. The process of inclusion can begin with an attitude of listening and engaging with challenging narratives.
